# Hydrocephalus Shunting in Supratentorial Glioblastoma: Functional Outcomes and Management

**DOI:** 10.3389/fonc.2022.796105

**Published:** 2022-02-09

**Authors:** Amir El Rahal, Debora Cipriani, Christian Fung, Marc Hohenhaus, Lukas Sveikata, Jakob Straehle, Mukesch Johannes Shah, Henrik Dieter Heiland, Jürgen Beck, Oliver Schnell

**Affiliations:** ^1^ Department of Neurosurgery, Medical Center - University of Freiburg, Freiburg im Breisgau, Germany; ^2^ Department of Neurosurgery, Department of Clinical Neurosciences, Geneva University Hospitals, Faculty of Medicine, Geneva, Switzerland; ^3^ J.P. Kistler Stroke Research Center, Department of Neurology, Massachusetts General Hospital, Harvard Medical School, Boston, MA, United States; ^4^ Division of Neurology, Department of Clinical Neurosciences, Geneva University Hospitals, Geneva, Switzerland

**Keywords:** glioblastoma, hydrocephalus, shunt, risk factors, quality of life, outcome, overall survival, KPS = karnofsky performance scale

## Abstract

**Background:**

Glioblastoma is the most common and the most challenging to treat adult primary central nervous system tumor. Although modern management strategies modestly improved the overall survival, the prognosis remains dismal associated with poor life quality and the clinical course often dotted by treatment side effects and cognitive decline. Functional deterioration might be caused by obstructive or communicating hydrocephalus but due to poor overall prognosis surgical treatment options are often limited and its optimal management strategies remain elusive. We aimed to investigate risk factors, treatment options and outcomes for tumor-associated hydrocephalus in a contemporary 10 years cohort of glioblastoma patients.

**Methods:**

We reviewed electronic health records of 1800 glioblastoma patients operated at the Department of Neurosurgery, Medical Center – University of Freiburg from 2009 to 2019. Demographics, clinical characteristics and radiological features were analyzed. Univariate analysis for nominal variables was performed either by Fisher’s exact test or Chi-square test, as appropriate.

**Results:**

We identified 39 glioblastoma patients with symptomatic communicating hydrocephalus treated by ventricular shunting (incidence 2.1%). Opening of the ventricular system during a previous tumor resection was associated with symptomatic hydrocephalus (p<0.05). There was also a trend toward location (frontal and temporal) and larger tumor volume. Number of craniotomies before shunting was not considered as a risk factor. Shunting improved hydrocephalus symptoms in 95% of the patients and Karnofsky Performance Score (KPS) could be restored after shunting. Of note, 75% of the patients had a post-shunting oncological treatment such as radiotherapy or chemotherapy, most prevalently chemotherapy. Infection (7.7%) and over- or under drainage (17.9%) were the most common complications requiring shunt revision in ten patients (25.6%), No peritoneal metastasis was found. The median overall survival (OS) was 385 days and the median post shunting survival was 135 days.

**Conclusion:**

Ventricular system opening was identified as a risk factor for communicating hydrocephalus in glioblastoma patients. Although glioblastoma treatment remains challenging, shunting improved hydrocephalus-related functional status and may be considered even in a palliative setting for symptom relief.

## Introduction

Glioblastoma multiforme (GBM) is the most common and deadly malignant central nervous system (CNS) tumor. GBM accounts for 48.6% of CNS tumors with an estimated incidence of 3.23 per 100’000 persons per year (1). Modern treatment strategies have improved overall prognosis; however, clinical course is often marked by significant treatment side effects, functional or cognitive decline. The current standard of care is maximal safe resection of the contrast-enhancing tumor followed by adjuvant radiotherapy and temozolomide chemotherapy (2). The overall survival rate for GBM patients improved from 3.3 months up to a median of 15 months in the past 30 years (3–8).

Moreover, tumor progression or complications occurring during the disease’s course can lead to neurologic deterioration such as hemiparesis, aphasia, cognitive decline, or gait disturbance, reflected by a reduction in the Karnofsky performance status (KPS) (9). Thus, despite increased life expectancy GBM patients often experience poor quality of life during the course of the disease (1, 3, 4, 7, 8).

Communicating hydrocephalus (CH) is a common complication during GBM course and can be readily detected in the presence of ventriculomegaly. However, often CH presents in an insidious fashion presenting with subacute cognitive decline, gait disturbance, or incontinence, overshadowed by prominent GBM-related symptoms or deficits. In addition, ventriculomegaly is challenging to identify in the context of treatment-associated cerebromalacia (10–12). The pathophysiology underlying GBM-related CH remains elusive with possible mechanistic explanations including cerebrospinal liquid circulation impairment due to ventricular opening, multiple surgical interventions, leptomeningeal metastases, and impaired CSF resorption due to radiotherapy-induced fibrosis, as well as tumor location (9, 11–15). Moreover, it remains unclear which symptoms are most likely to improve after shunting of glioblastoma patients and what would be the best time point to intervene in this situation. Our study aimed to investigate the risk factors, treatment options and functional outcomes for tumor-associated hydrocephalus and post-shunting oncological therapy in a contemporary 10 years cohort of GBM patients.

## Material and Methods

### Patient Data Acquisition

We retrospectively reviewed all GBM patients treated at the Department of Neurosurgery, Medical Center – University of Freiburg from January 2009 to December 2019 following the STROBE statement and guidelines (16). Out of 1800 glioblastoma patients in total, we identified 39 patients presenting a communicating hydrocephalus. Inclusion criteria were: 1) Histologically confirmed GBM and available molecular profiling 2) Suspected symptomatic hydrocephalus, 3) age of 18 years or older, 4) at least one previously attempted complete resection, 5) available pre- and post-operative MRI within 72 hours and for follow-up. All cases were treated by ventriculo-peritoneal (VP) or ventriculo-atrial (VA) shunting.

Clinical data were extracted from electronic medical records. We collected the following clinical variables: 1) pre-operative KPS 6-12 weeks before surgery and at admission, 2) KPS 6-12 weeks after shunting, 3) date of last follow-up, 4) death date.

We collected the following tumor- and surgery-related variables: location, tumor volume (cm^3^), ventricular opening during previous tumor surgery and leptomeningeal spreading.

Written informed consent was obtained from all patients. Ethical approval was obtained from the local ethics committee (Freiburg ethic commission N: 21-1272). Demographics and clinical characteristics are presented in **Table 1**. Four patients with obstructive hydrocephalus or a loss of follow-up were excluded from the study.

**Table 1:** Patient’s demographics and admission parameters.

Patient Demographics	N = 39	%
Gender		
Female	13	33.3
Male	26	66.6
Age in years		
Median (IQR)	56.1 (46.5.7-62.8)	
GSC at admission		
Median (IQR)	14 (13-15)	
Hydrocephalus-related symptoms		
Gait disturbance	36	92.3
Headache	33	84.6
Cognitive decline	28	71.8
Incontinence	13	33.3
Motor deficit	12	30.7
Hakim’s Triad	10	25.6
MGMT – Promoter status methylation		
Non methylated	20	51.3
Methylated	5	12.8
NA	14	35.9

Males were predominant in our cohort, and the median age was 56.1 years. Gait disturbance is the most prevalent symptom and Hakim’s triad is present in approximately 25% of patients on admission. IQR, Interquartile range; MGMT, Promoter status methylation; NA, Not available.

### Histopathological and Molecular Analysis

Diagnosis of GBM was based on the 2016 WHO Classification of Tumors of the Central Nervous System (17). Specimens were analyzed using the standard protocol at the Institute of Neuropathology, Medical Center-University of Freiburg as described in previous publications (17–19). IDH mutations were detected by immunohistochemistry (IHC). In patients <65 years old, next-generation sequencing of IDH1 and IDH-2 was performed to confirm negative staining results. MGMT promoter methylation status was performed using methylation-specific PCR.

### Hydrocephalus Ascertainment

Communicating hydrocephalus was suspected in the setting of emergent ventricular enlargement and associated clinical symptoms. Hydrocephalus-associated clinical symptoms were collected: headache, cognitive decline, gait disturbance, or urinary incontinence. The latter three symptoms comprising the Hakim’s triad (20). We calculated the Evans’s ratio based on the preoperative MRI or CT (21). In brief, Evan’s index is the ratio of the maximum width of the frontal horns of the lateral ventricles and the maximal internal diameter of the skull at the same level. Evan’s index of >0.3 was indicative of hydrocephalus. A lumbar tap test was routinely performed to evaluate post-tap clinical improvement When hydrocephalus was diagnosed, a shunt was placed with a MiniNav 10® valve or proGav 2.0® by Miethke valve and occasionally other type of valves (Dual Switch 5/30® or Dual Switch 10/30® by Miethke) either by a ventriculo-peritoneal (VP) or ventriculo-atrial (VA) shunt procedure.

### MR Imaging Acquisition

MRI acquisition was realized on 1.5 or 3.0 Tesla whole body system. Anatomical imaging used for resection analysis consisted of 3D T1-weighted sequences before and after contrast application. Patients usually received a preoperative and postoperative MRI within 48-72h and every 3 consecutive months. Gross total resection was defined as removal of more than 95% of the contrast-enhancing tumor (22). Tumor progression was defined according to the RANO-criteria (23). An emphasis was placed on the following factors: leptomeningeal tumor spreading, ventricular wall enhancement, and tumor location. The volumetric segmentation of the tumor was performed using the Elements software proposed by BrainLAB^®^. Tumor volume was measured in cm3.

### Oncological Treatment

Patients were treated according to the standard of care protocol by Stupp et al. in 2005 (2). In brief, patients underwent gross total resection of contrast-enhancing tumor, adjuvant radiotherapy and temozolomide chemotherapy. In some cases, patients received alternative chemotherapeutic treatments (lomustine), antiangiogenic therapy with bevacizumab, or radiotherapy alone. Intraoperative chemotherapeutics such as BCNU (Carmustine) wafers were not administered to any of the 39 patients.

### Study Endpoints

The primary endpoint was the clinical and functional outcome of patients benefiting from shunting for a communicating hydrocephalus. For this purpose, we measured the KPS and collected variables related to clinical symptoms and parameters.

The secondary endpoints were 1) the clinical symptoms experienced, 2) overall survival in GBM shunted patients, and 3) the median postoperative survival time after the shunt placement.

### Statistical Analysis

Statistical analysis was performed using R software [version R 4.0.4] through the studio interface Version 1.4.1106. Univariate analysis for nominal variables was performed either by Fisher’s exact test or Chi-square test, as appropriate. Results are reported as odds ratio with 95% confidence intervals and 2-sided p values. The statistical differences were considered significant at a p<0.05. Bonferroni correction was used to account for type I error when conducting multiple analyses on the same dependent variable. Kaplan-Meier analysis was used to estimate the survival distributions. Patients’ loss of follow-up were censored at the recorded date of last contact or consultation.

### Illustrative Case

**Figure 1:**
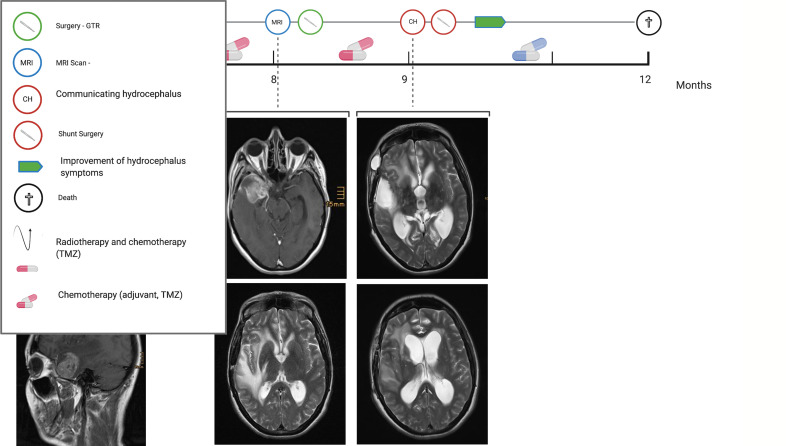
Illustrative case of a 50 y/o woman with a right temporal GBM WHO grade IV, IDH wildtype and unmethylated MGMT promoter. Eight months after the first resection, the patient presented a recurrence with a second surgery performed and a repeated GTR achieved. One month later patient presented clinically and radiological a communicating hydrocephalus requiring shunt. Unfortunately, the patient died after 12 months. Created with Biorender.

We illustrate in **Figure 1** the case of a 50 years old woman known for non-structural epilepsy since her childhood treated with Carbamazepine. She presented with new symptoms including hallucinations, headaches, and fatigue. She consulted at the neurosurgical department and an MRI showed a left temporal contrast-enhancing tumor. The first resection was performed the same month with a gross total resection (GTR) and no contrast-enhancing residual lesions were seen on the postoperative MRI. The histopathological analysis revealed a glioblastoma WHO IV, IDH wildtype with unmethylated MGMT promoter. Radiochemotherapy according to the Stupp protocol was introduced without complications (2).

After 8 months she developed a tumor recurrence. A second surgery with GTR was achieved and the temporal horn was opened during the procedure. One month later she presented new symptoms with acute drowsiness (GCS 12 on admission), gait disturbance, cognitive decline, and incontinence compatible with the Hakim’s triad. The MRI revealed a ventriculomegaly with Evan’s Ratio of >1. A lumbar puncture revealed a high level of protein >1.5g/l and the patient improved clinically after the lumbar puncture A VP shunt was implemented with a Miethke MiniNav 10® valve. The patient improved clinically with resolving symptoms and an increase in KPS from 40 to 50 post-operatively. This strategy allowed the patient to maintain her quality of life and successfully receive chemotherapeutic treatment due to an improved functional status. Despite the treatment, the patient died three months later.

## Results

### Patient Characteristics

**Figure 2:**
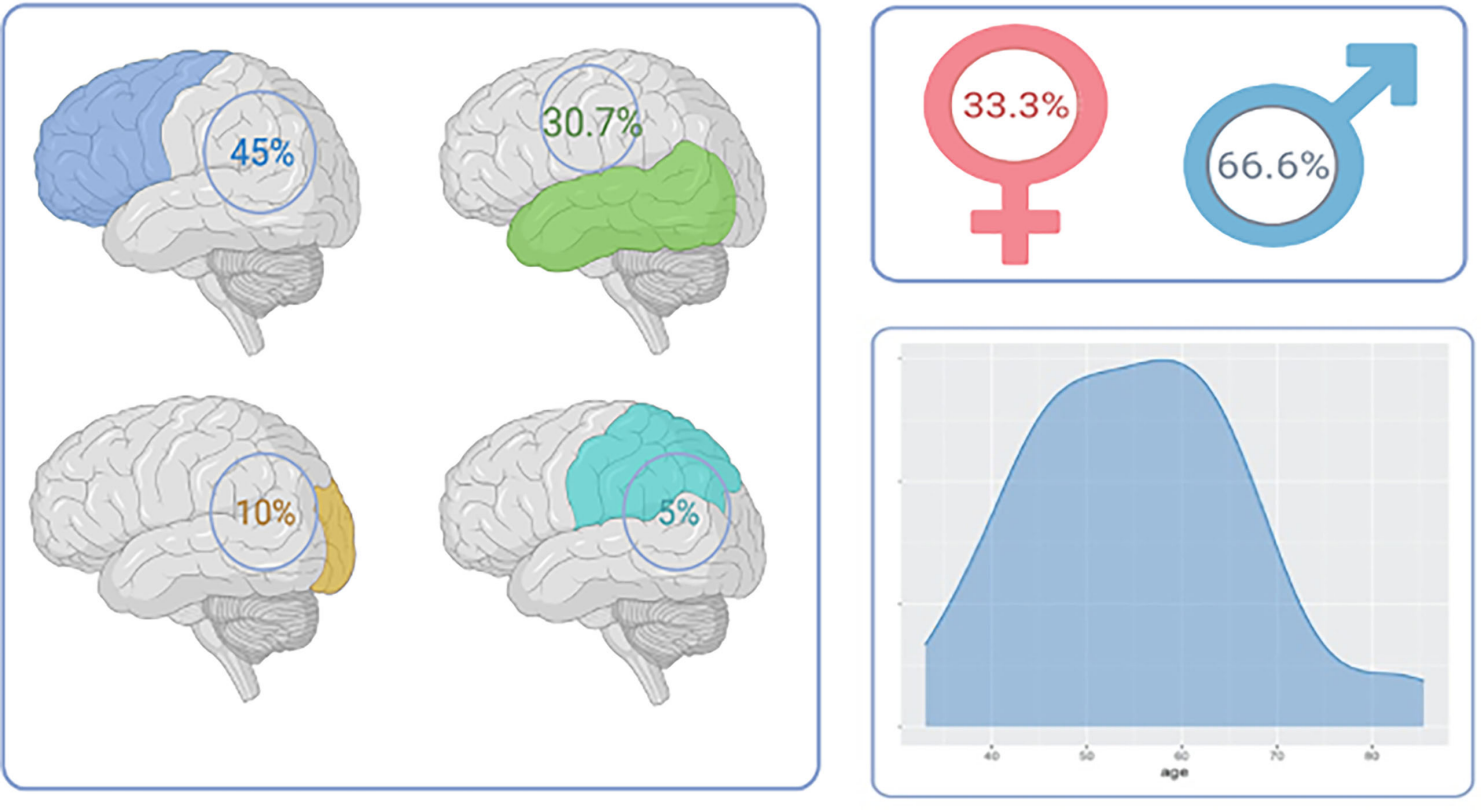
Tumor location and patient demographics in GBM-related hydrocephalus cohort. Left panel: Tumor location showing a predominance of GBM in the frontal lobe followed by temporal tumors. Right upper panel: sex distribution. Right lower: age distribution.

Thirty-nine patients were treated surgically for a supratentorial glioblastoma multiforme WHO grade IV and benefited from a shunt for communicating hydrocephalus (CH) at the Medical Center-University of Freiburg between 2009 and 2019. The median age was 56.1 years (IQR 46.5. - 62.8), 66,6% were male and 33% were female (**Figure 2**). The mean time between the first tumor resection to shunt placement was 187 days (IQR 45.5 – 176.5). Among 25 patients with measurements of the MGMT methylation status, we found the methylation to be present in 5 patients (12.8%) and absent in 20 patients (51.2%) (**Table 1**).

### Treatment

A gross total resection was attempted in all patients. Most patients underwent a single surgery before shunt placement 17/39 (43.5%), 14 patients underwent two resections (35.9%), and 8 patients had three or four resections (20.5%). Chemotherapy was given in 37 (95%). Among patients treated with chemotherapy, all were treated with temozolomide. In addition to temozolomide, six patients (15%) were treated with bevacizumab before shunt treatment. No patients benefited from intracavity BCNU wafers. Radiotherapy was also performed in 37 (95%) patients. One patient had an early glioblastoma recurrence before any adjuvant treatment could be started, and one was lost to follow-up. Of note, 75% of the patients had a post-shunting oncological treatment such as radiotherapy or chemotherapy, most prevalently chemotherapy.

### Diagnostics Features of Patients With Communicating Hydrocephalus

Symptoms preceding the clinical or radiological diagnostic of hydrocephalus were gait disturbance in 35 (90%), headaches in 33 (85%), cognitive decline in 28 (72%). Only 10 (25.6%) presented with the typical Hakim’s triad. Median GCS on admission was 14 (IQR 13-15). Nine (23%) patients presented acute drowsiness related to hydrocephalus.

Eighteen (46%) patients received a lumbar tap test where 20-40 ml were withdrawn which resulted in a transient improvement of symptoms in all patients. CSF protein concentration levels were only analyzed in half of the cases and therefore couldn’t be interpreted. Regarding the post resection compilations, seven (18%) patients had a CSF leak (18%), six patients had a postoperative complication such as meningitis in 3 patients and 3 suffered from a postoperative hemorrhage (6.6%).

### Radiological Characteristics

The frontal lobe tumor location was present in 45% followed by the temporal lobe in 30.7%, occipital lobe in 10%, and parietal lobe in 5%. In 12.5% of the cases, a cortico-subcortical tumor invading the deep structures was diagnosed. Thirty-two (82%) patients received a gross-total resection, 4 patients had between 70-90% of the tumor resected and 4 less than 50% of the tumor resection (**Figure 2**).

Evans’ index of > 0.3 as mentioned above, considered as positive and was found in 20 patients out of 39 at diagnosis (51%) with no statistical correlation as an independent risk factor (p>0.05).

### Treatment for CSF Diversion

All patients received a VP shunt in the first intention. A differential non-adjustable pressure valve (Miethke MiniNAV 10) was implanted in 25 (64%), an adjustable valve (Miethke proGAV) in 9 (23%), and other valves in 5 patients (usually Dual Switch 5/30^®^ and Dual Switch 10/30^®^).

### Shunt Implantation Outcomes

Ten patients (26%) with implanted shunts required a revision surgery. Among patients requiring a revision, in three patients (30%) it was due to an early (<30 days) and seven (70%) due to a late shunt complication (**Table 2**). In the early complication group, two had an infection and one an early shunt malfunction. In the late complication group, there was one case of infection and six cases of valve malfunction (over- or under drainage). No peritoneal metastases were found in the whole cohort.

**Table 2:** Shunt complications requiring revision surgery.

Shunt complications requiring revision surgery	N = 10	25.6%
**Infection**	**3**	**7.7%**
• Early < 30 days	2	
• Late >30 days	1	
**Malfunction**	**7**	**17.9%**
• Early < 30 days	1	
• Late >30 days	6	

In toto 10 patients required a revision surgery with “3 infection and 7 shunt dysfunctions” representing respectively the bold values in column 1 and column 2.

### Risk Factors for HC in Glioma Patients

Ventricular system opening was associated with hydrocephalus Chi square test p<0.05). The number of craniotomies, tumor volume or localization were not associated with hydrocephalus. Leptomeningeal enhancement was found in 8 (20.5%) patients and was not associated with hydrocephalus.

### Postoperative Clinical Performance and GBM Survival

**Figure 3:**
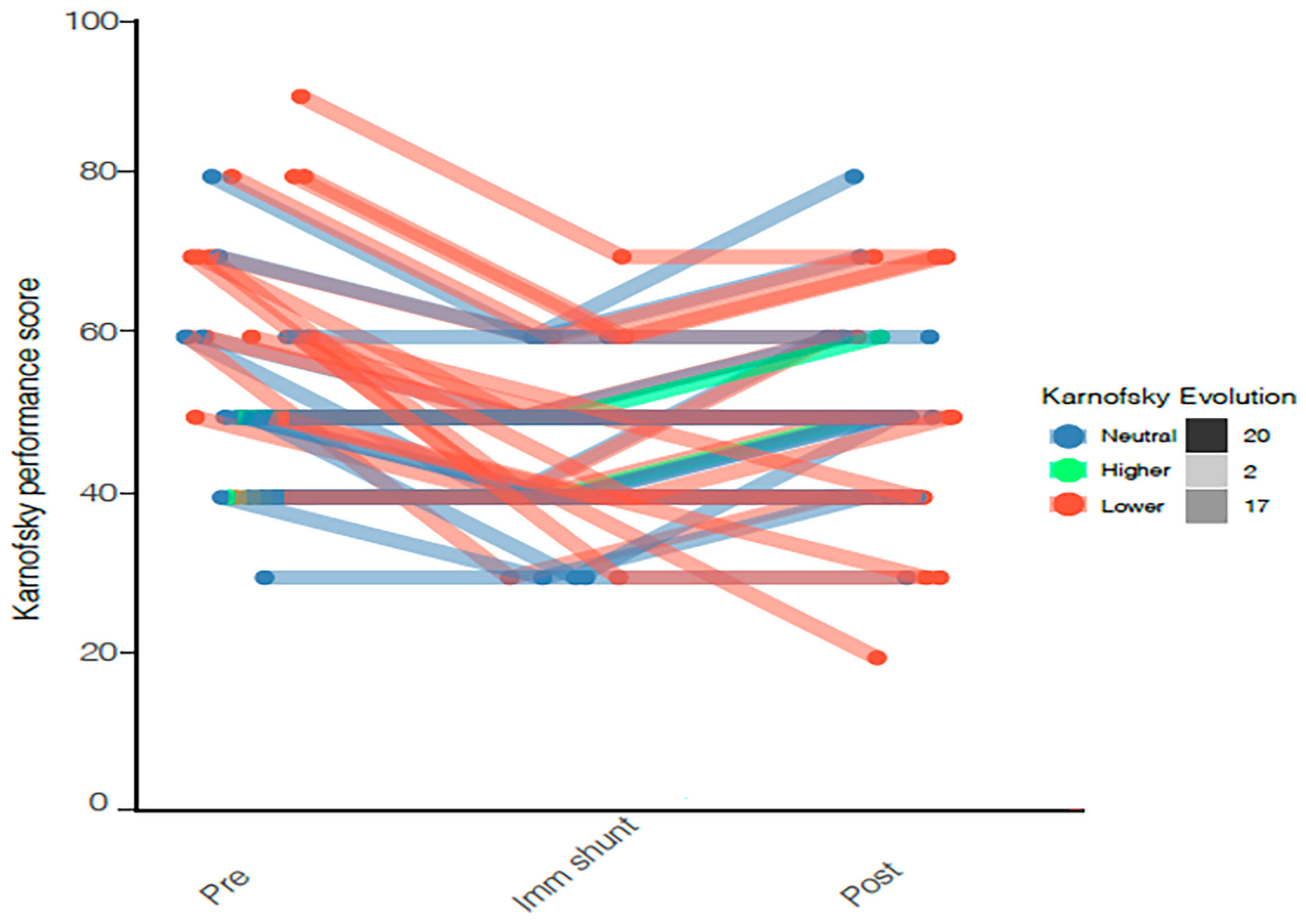
KPS before, immediately before shunting, and after shunting is represented by a Line plot showing individual KPS. Progression in the KPS is colored in green, a decline in red and stability in blue. Median KPS before and after surgery is 50 with no statistical difference.

Thirty-seven (95%) patients had a symptomatic improvement after shunting. Of the other two patients, one died shortly after shunting and the second one was lost to follow up. The median of the last documented KPS during neuro-oncological routine follow-up before shunting was 50 (IQR 30-65). However, immediately before shunting, a dip was observed revealing a median of 40 (IQR 30-50). Finally, the median KPS post-op (6-12 weeks) was 50 (IQR 40-60) again. Therefore, no statistical difference was found when comparing the KPS 6-12 before and KPS 6-12 weeks after surgery but the acute deterioration before shunting was indicated led to a dip in the KPS of patients reflecting their general status (**Figure 3**).

**Figure 4:**
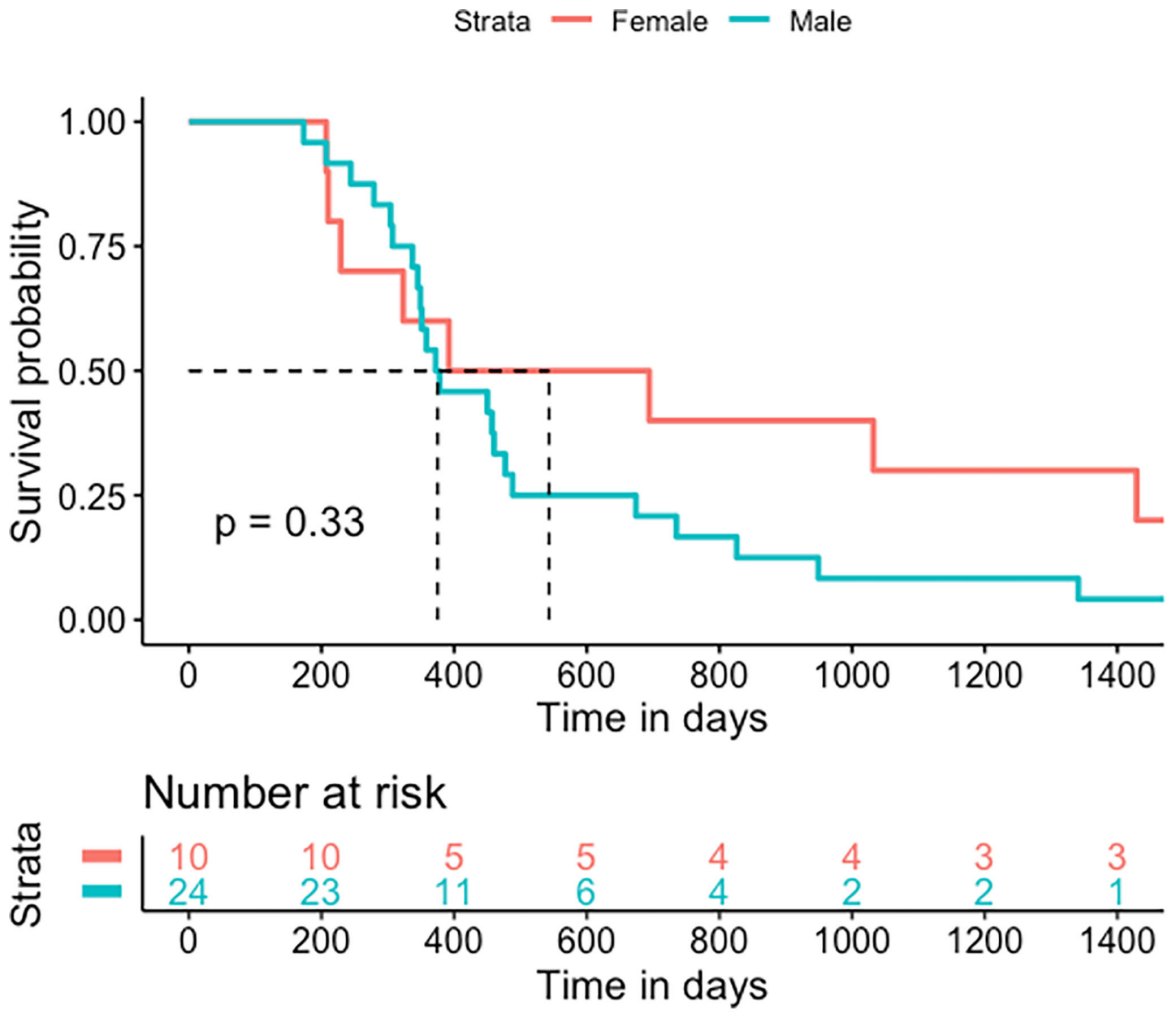
Kaplan-Meier statistics for the overall survival of GBS patients treated for hydrocephalus. Median OS was 385 days (IQR 311-724).

**Figure 5:**
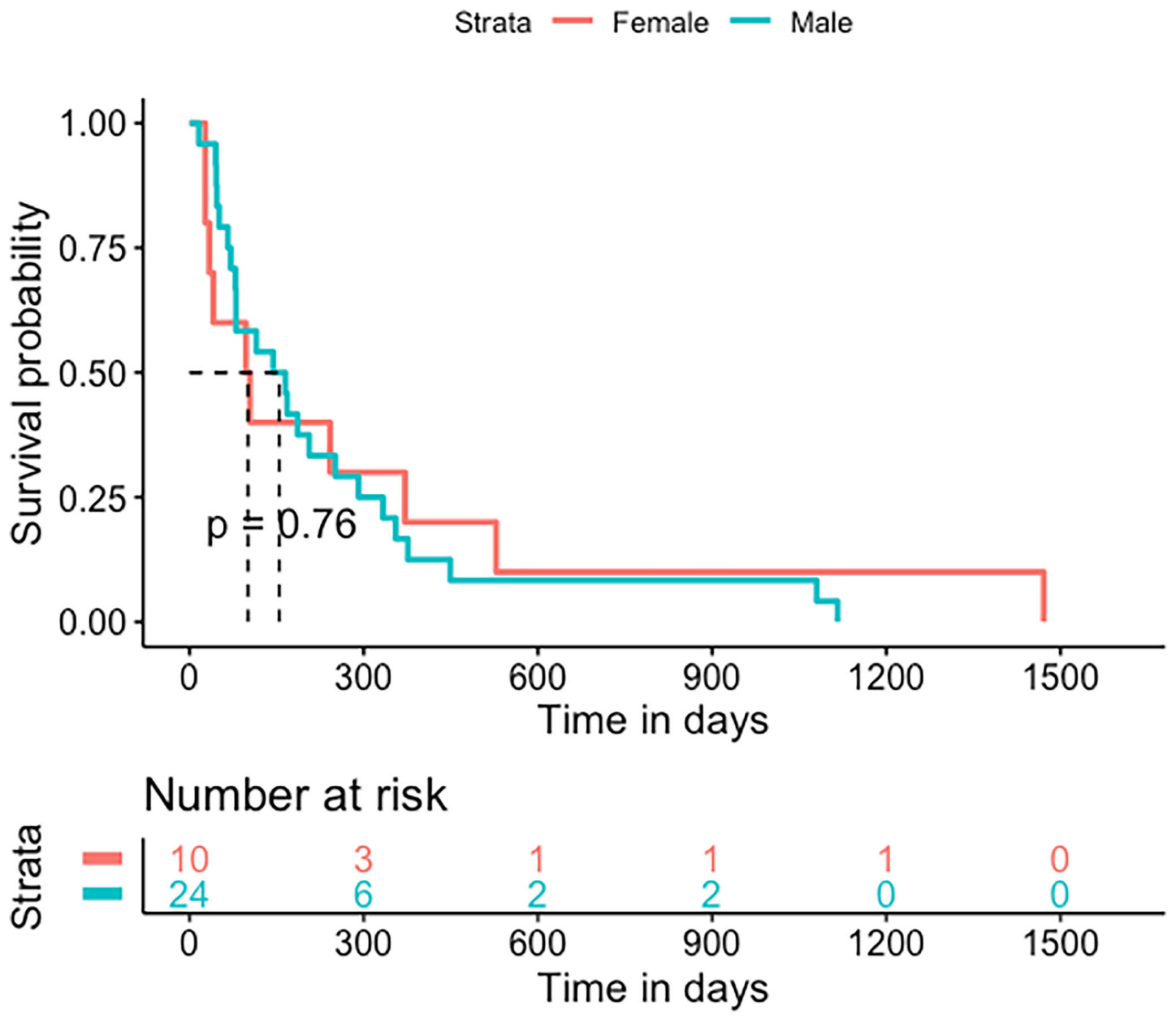
Kaplan-Meier statistics for post-shunt survival. Shunt to death median survival was 130 days (IQR 54.75-322).

The OS after GBM diagnosis was 385 days (IQR 311-724) (**Figure 4**). The median shunt to death survival was 130 days (IQR 54.75-322) (**Figure 5**).

## Discussion

Modern treatment concepts have significantly improved the overall prognosis for glioblastoma patients during the past three decades (3–8). However, this gain in life expectancy did not lead to the same extent of improvement in quality of life in these patients (24–26). Therefore, a paradigm shift towards focusing on factors contributing to improvement, maintenance or decline of quality of life might be necessary in order to help our patients benefit from novel therapies and multi-modal management.

The incidence of post-operative communicating hydrocephalus has been estimated to range between 2 and 10% consistent with our findings of 2.1% (9–11, 13–15, 27, 28). The mechanisms responsible for communicating hydrocephalus in the context of glioblastoma surgery are not entirely understood and few studies have addressed this underrecognized issue. The usually presumed mechanism is leptomeningeal tumor cell dissemination that impairs CSF absorption, proteinic precipitation, or fibrosis of arachnoid granulations due to radiation. Therefore hydrocephalus management is still a matter of debate in glioblastoma patients (10–14, 29, 30) and the decision-making process needs a personalized approach. With one of the biggest series in literature (10–14, 27, 29, 30), we provide evidence that although shunting may not prolong overall survival, it may help improve symptoms and functional performance of patients. This is illustrated by maintained KPS after shunting reflecting stability in the daily quality of life of patients.

Glioblastoma patients usually develop cognitive decline due to tumor progression, radiation-induced brain atrophy, CSF tumor dissemination, seizures, or even general condition alteration (31–34). CSF disturbance might be a contributing factor of clinical deterioration and treatment by VP or VA shunt can reverse or stabilize the general condition of patients, as shown in our cohort.

Improvement of symptoms after shunting was reported to vary between 61% and 100% (9, 12, 13, 29, 30, 35). With a 95% improvement rate after shunting our findings align with these previous reports. Interestingly, we observed two different aspects regarding the general condition of patients. First, there was a dip in the KPS just before shunting, returning to baseline after shunting. Second, more than 75% of the patients could benefit from chemotherapy and radiotherapy post-shunting. We demonstrate that the hydrocephalus-related clinical decline was reversed by shunting and helped maintain patients’ clinical condition. We can also assume that shunting may prevent further clinical deterioration by halting the hydrocephalus symptomatology progression. Thus, we conclude that shunt placement should not be delayed since there might be a threshold beyond which some of the symptoms may not be fully reversible. Furthermore, worse functional status precludes oncological treatment and might shorten the overall survival. We continue to need better preoperative screening and indicators to determine which patients will benefit from shunting. Strategy implicating new management like infusion test might be a useful tool in the future (36, 37).

Historically, one of the major concern of shunt implantation in glioblastoma patients is the risk of peritoneal metastasis (32, 38–41). However, it is now well established that spread by shunts is a rare albeit potential serious complication in high-grade gliomas. However, in our study, no patients presented with peritoneal metastasis. This confirms the hypothesis that peritoneal metastasis is a rare complication and might not be a major obstacle for hydrocephalus treatment in GBM.

The Karnofsky performance score is a well-established score that is simple to use and has been validated in the functional evaluation of oncological patients (42). Nevertheless, limitations were noted regarding its adequacy for quality of life evaluation (43, 44). New scores were developed, such as the NANO score, which promises better accuracy in estimating neurologic function and, therefore, life expectancy, but still needs to be validated regarding the quality of life (45). Whether shunting positively influences overall survival or whether this leads to an improvement in QoL would need to be validated in a prospective study. In fact, we neither have a control group nor a structured QoL questionnaire due to the retrospective nature of our study.

The delicate balance between a shunting procedure to relieve symptoms and the overall survival in glioblastoma has to be considered in the context of an optimal neuro-oncological treatment. The surgical complication rate in our series was acceptable with ten patients requiring revision surgery (25%) of whom three patients (7.7%) had an early complication (<30 days) and seven (17.9%) a late complication. Interestingly, no major complications were encountered. This is in line with the findings of Castro et al. where 29% of complications were reported without any major complication (30). Roth et al. reported in 2008 a rate of complication of 50% with a rate of 33% of infections and major events such as coma or death in 12.5% (12). This is also in line with rates previously reported by Giordan et al. in a recent review regarding shunting in idiopathic normal pressure hydrocephalus (46). As a matter of fact, the revision rate reported in this meta-analysis is about 18% regarding a shunt malfunction, similar to our dysfunction revision rate of 17.9% (46). Life expectancy was not affected by shunt revision in our cohort. Therefore, we conclude that shunting complication risk should not be a reason to defer shunting. Patients with acute clinical decline without radiological findings of tumor progression and with signs or symptoms of hydrocephalus should be considered for a shunt placement. In most cases, shunt placement led to a reversal of the acute deterioration presented by an acute dip on the KPS.

### Limitations and Strengths

Although, our work is based on retrospective analysis, it provides data supporting an important feature in GBM patients which is quality of life and palliative support. Compared to other studies our cohort included only CH in GBM and this limits biases caused by mixed hydrocephalus etiology. Our cohort is one of the biggest published recently even if limited by the small number of patients allowing limited analysis of risk factors of CH in the context of a GBM.

## Conclusion

Treatment of hydrocephalus in the context of a glioblastoma is challenging but improves symptoms in most patients and may therefore be considered in routine care and in a palliative setting for relief of symptoms. The benefit of symptomatic improvement is higher than the complication and morbidity rate linked to shunting. We conclude that early detection of CH might maintain patients’ eligibility for crucial oncological therapy as well as quality of life. Novel strategies are warranted to improve the early detection of glioblastoma-related hydrocephalus.

## Data Availability Statement

Datasets are available from the corresponding author or the senior author on reasonable request.

## Ethics Statement

The studies involving human participants were reviewed and approved by Ethical committee Freiburg im Breisgau. (Freiburg ethic commission N: 21-1272). informed consent for participation was not required for this study in accordance with the national legislation and the institutional requirements. Written informed consent was obtained from the individual(s) for the publication of any potentially identifiable images or data included in this article.

## Author Contributions

AER and OS contributed to conception and design of the study. AER, DC, MH, HH, and JS organized the database. AER and HH performed the statistical analysis. AER and OS wrote the first draft of the manuscript. CF wrote sections of the manuscript. LS, MHS and JB provided substantial corrections to the final manuscript. All authors contributed to the article and approved the submitted version.

## Funding

AER received a fellowship grant from The Nuovo-Soldati oncology research foundation, Vaduz, Liechtenstein. JS received funding from the Berta-Ottenstein-Programme for Clinician Scientists, Faculty of Medicine, University of Freiburg, Germany. HH is funded by the Else Kröner- Fresenius Foundation.

## Conflict of Interest

The authors declare that the research was conducted in the absence of any commercial or financial relationships that could be construed as a potential conflict of interest.

## Publisher’s Note

All claims expressed in this article are solely those of the authors and do not necessarily represent those of their affiliated organizations, or those of the publisher, the editors and the reviewers. Any product that may be evaluated in this article, or claim that may be made by its manufacturer, is not guaranteed or endorsed by the publisher.

## Abbreviations

GBM, Glioblastoma multiforme; KPS, Karnofsky performance score; CNS, Central nervous system; OS, Overall survival; MR, Magnetic resonance; GTR, Gross total resection.
